# Dual attentive fusion for EEG-based brain-computer interfaces

**DOI:** 10.3389/fnins.2022.1044631

**Published:** 2022-11-23

**Authors:** Yuanhua Du, Jian Huang, Xiuyu Huang, Kaibo Shi, Nan Zhou

**Affiliations:** ^1^College of Applied Mathematics, Chengdu University of Information Technology, Chengdu, China; ^2^Centre for Smart Health, The Hong Kong Polytechnic University, Hong Kong, Hong Kong SAR, China; ^3^School of Electronic Information and Electronic Engineering, Chengdu University, Chengdu, China

**Keywords:** brain-computer interface, electroencephalography, P300, motor imagery, dual attentive fusion

## Abstract

The classification based on Electroencephalogram (EEG) is a challenging task in the brain-computer interface (BCI) field due to data with a low signal-to-noise ratio. Most current deep learning based studies in this challenge focus on designing a desired convolutional neural network (CNN) to learn and classify the raw EEG signals. However, only CNN itself may not capture the highly discriminative patterns of EEG due to a lack of exploration of attentive spatial and temporal dynamics. To improve information utilization, this study proposes a Dual Attentive Fusion Model (DAFM) for the EEG-based BCI. DAFM is employed to capture the spatial and temporal information by modeling the interdependencies between the features from the EEG signals. To our best knowledge, our method is the first to fuse spatial and temporal dimensions in an interactive attention module. This module improves the expression ability of the extracted features. Extensive experiments implemented on four publicly available datasets demonstrate that our method outperforms state-of-the-art methods. Meanwhile, this work also indicates the effectiveness of Dual Attentive Fusion Module.

## 1. Introduction

Brain-computer interface (BCI) is a system that aims to establish a non-muscular communication pathway between humans and external devices *via* brain signals (Wolpaw et al., 2002). With the advances in information and computer science, various BCI paradigms have been developed and employed in many applications (Leeb et al., 2007; Dal Seno et al., 2010). The motor imagery (MI) paradigm attracts significant interest from researchers. It is the process of imagining movement in a certain body part rather than actually moving it. This technology can help patients with movement disorders manipulate external equipment such as artificial arms or wheelchairs (Leeb et al., 2007). The P300 event-related potential (ERP) is also another important paradigm in BCI. It is an evoked positive peak at around 300 ms after the occurrence of a low-probability stimulus. This type of BCI has been utilized to assist individuals with severe neuromuscular diseases to spell characters by using brain waves (Dal Seno et al., 2010). Currently, electroencephalography (EEG) is one of the most widely used techniques for brain signal acquisition in BCIs due to its low cost, safety, and easy operation. The core of EEG-based BCI is to analyze EEG signals for the purpose of understanding human intentions. Therefore, improving the performance of EEG-based BCIs is very important for the future development of BCIs.

Generally, the classification based EEG first extracts discriminative features from EEG signals and adopts classifiers to classify the extracted features. However, it is not easy to deploy these processes due to the low signal-to-noise ratio (SNR) of EEG signals. Many previous methods rely on feature engineering and traditional machine learning approaches. For example, Rakotomamonjy and Guigue (2008) used a P300 detection model based on 896 hand-crafted features and an ensemble of SVMs classifiers. Fazli et al. (2009) proposed to combine feature extraction from the common spatial pattern (CSP) of the EEG signals and linear discriminant analysis (LDA) to classify the extracted features. The method developed by Li et al. (2006) alternatively used independent component analysis (ICA) to remove eye artifacts and selected a subset of electrodes prior to the classification made by support vector machine (SVM). Duan et al. (2013) first combined an SVM and K-nearest neighbor (KNN) to extract and classify features from multi-channel EEG data for emotion recognition. In Liu et al. (2005), principal component analysis (PCA) and T-weight value sums were applied for P300 classification. Although these attempts have achieved partial improvements in performance, all these methods only learn the features that the researchers focus on while ignoring other important features due to the limited abilities of hand-crafted features.

In addition, considerable effort has also been devoted to developing deep learning (DL) based methods for EEG signal classification (Zhang Y. et al., 2020; Huang et al., 2021), and they have demonstrated superior performance over conventional machine learning methods. Especially due to the temporal dynamics of EEG signals, recurrent neural network (RNN) based methods have been extensively applied to filter and classify EEG signals (Alhagry et al., 2017; Ma et al., 2018; Michielli et al., 2019). Alhagry et al. (2017) used an LSTM-RNN to learn and classify EEG signals for emotion recognition. Ma et al. (2018) proposed a pure RNNs-based parallel method to encode spatial and temporal information of raw EEG signals for motor imagery classification. Michielli et al. (2019) introduced a novel cascaded RNN architecture based on long short-term memory (LSTM) blocks for automated sleep stage classification.

Apart from RNN, convolutional neural network (CNN) has been popularly used for analyzing EEG signals and has gained much attention in recent years (Lawhern et al., 2018; Sakhavi et al., 2018; Shan et al., 2018; Yang et al., 2018a; Wu et al., 2019; Ding et al., 2021). Lawhern et al. (2018) presented a compact neural network named EEGNet, which can extract spatial and temporal features simultaneously. Wu et al. (2019) proposed a parallel multi-scale filter bank CNN architecture, generating temporal, and spatial features for MI classification. Ding et al. (2021) proposed TSception, a multi-scale CNN that learns discriminative in the time and channel dimensions to recognize the BCI's user emotion. Convolutional recurrent neural network (C-RNN) (Yang et al., 2018b; Zhang et al., 2018) was applied in EEG-based BCI and attained satisfactory performance. For example, Zhang et al. (2018) introduced cascade and parallel C-RNN models for human intention recognition and effectively learned the spatial-temporal representations of raw EEG signals. All these studies show the information in spatial and temporal dimensions carrying important information for BCI classification tasks. However, previous architectures handle the information of the EEG signal in temporal and spatial dimensions in either separate or subsequent manner without interaction.

Corresponding to this gap, we propose a simple but effective Dual Attentive Fusion Model (DAFM) for the EEG signal classification tasks. It leverages an interacting mechanism, which fuses spatial and temporal attention with a simple operation to generate the spatial-temporal pattern of the EEG signals. The main contributions of this paper can be summarized as follows.

1. The proposed model uses an interactive attention module, which can take both the spatial and temporal dimensions into consideration, and it successfully derives distinguishable features from EEG signals.
2. The proposed method is extensively evaluated on four widely used BCI datasets regarding both motor imagery (MI) and P300 tasks. Results exhibit that our approach has superior performance to state-of-the-art and baseline methods.

The remaining of this paper is organized as follows. Related works are described in Section 2. Section 3 presents the proposed method. Section 4 provides the datasets used in this paper, implementation details of our experiments and experimental results. Finally, Section 5 concludes this study.

## 2. Related work

### 2.1. Convolutional neural network

In recent years, deep learning, especially Convolutional Neural Networks (CNNs), has gained substantial interest in the computer vision field (Krizhevsky et al., 2012; Simonyan and Zisserman, 2014; He et al., 2016). In 2012, Krizhevsky et al. (2012) proposed the AlexNet, which used a large, deep convolutional neural network to classify images in the ImageNet dataset and achieved considerably better results than the previous state-of-the-art methods. Convolutional frameworks have become an essential medium in vision-related fields. VGGNet proposed by Simonyan and Zisserman (2014) has good transfer learning ability. Since then, the 3 × 3 convolution has become the standard configuration of the subsequent convolutional neural network structures. In 2015, He et al. (2016) noticed the gradient vanishing problem caused by the deepening of the network, and proposed the ResNet, which got rid of the troubles of the deep network and made the network depth reach astonishing 152 layers. Recently various CNN-based models are increasingly being used for EEG-based BCI and gain excellent performance. Sakhavi et al. (2018) introduced a new temporal representation of the data and used a CNN architecture for MI classification. Shan et al. (2018) proposed a novel and simple CNN, which only used a convolutional layer, to effectively learn feature representations from both temporal and spatial information for accurate P300 detection. Yang et al. (2018a) proposed to combine features of signals from different frequency bands and used a continuous convolutional neural network to make predictions.

### 2.2. Attention mechanism

It could be said that the attention mechanism has become one of the hottest topics in the deep learning field. The attention mechanism, which can selectively amplify valuable features and suppress useless features based on global information, has been employed in diverse domains. Fu et al. (2019) proposed a novel Dual Attention Network (DANet) to capture feature dependencies in the spatial and channel dimensions for scene segmentation. Huang et al. (2019) proposed a novel Criss-Cross Network (CCNet) to capture full-image contextual information adaptively in a more efficient way for semantic segmentation. Chen et al. (2019) proposed an Attentive but Diverse Network (ABD-Net) to integrate attention mechanism into ABD-Net, containing Channel Attention Module, and Position Attention Module for person re-identification. The attention mechanism is also used to transform the input into a more discriminative representation in the brain-computer interface field. Kim and Choi (2020) combined an attention mechanism and a long short-term memory network to assign weights to different emotional states based on importance and improved emotion recognition accuracy. Tao et al. (2020) proposed an attention-based convolutional recurrent neural network (ACRNN), which integrated the channel-wise attention into CNN to extract spatial information and extended self-attention into RNN to extract temporal information. Zhang D. et al. (2020) proposed a Graph-based Convolutional Recurrent Attention Model (G-CRAM) to explore EEG features across different subjects for motor imagery classification. Graph structure was employed to enhance the discriminative ability of EEG channels in this model.

## 3. Methods

**Figure 1:**
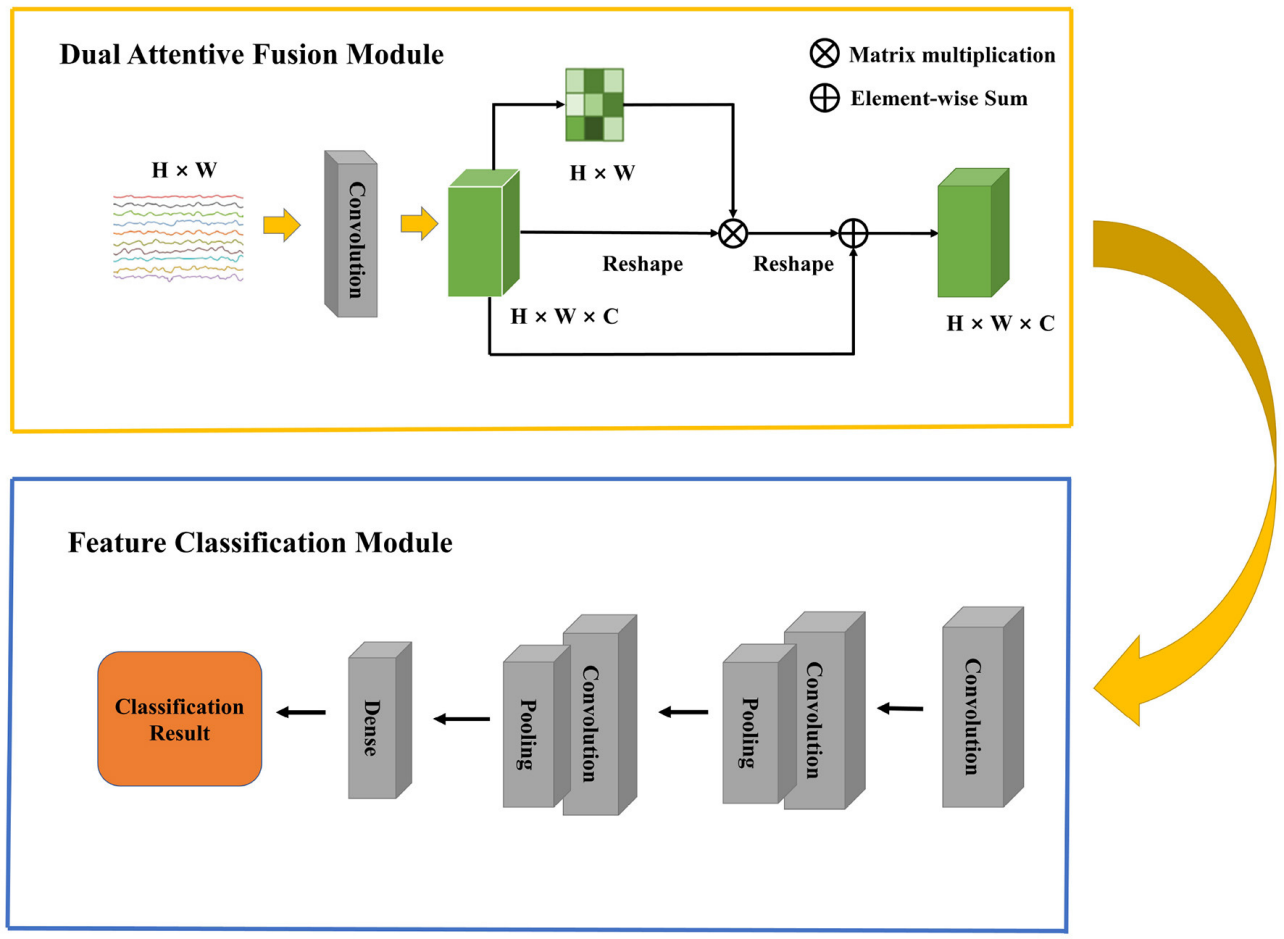
An overview of the Dual Attentive Fusion Model.

In this section, we first present an overall framework of our network, which contains two modules. Then, we describe the details of Dual Attentive Fusion Module. Finally, we introduce the Feature Classification Module. The overall architecture of our model is shown in Figure 1.

### 3.1. Overview

**Figure 2:**
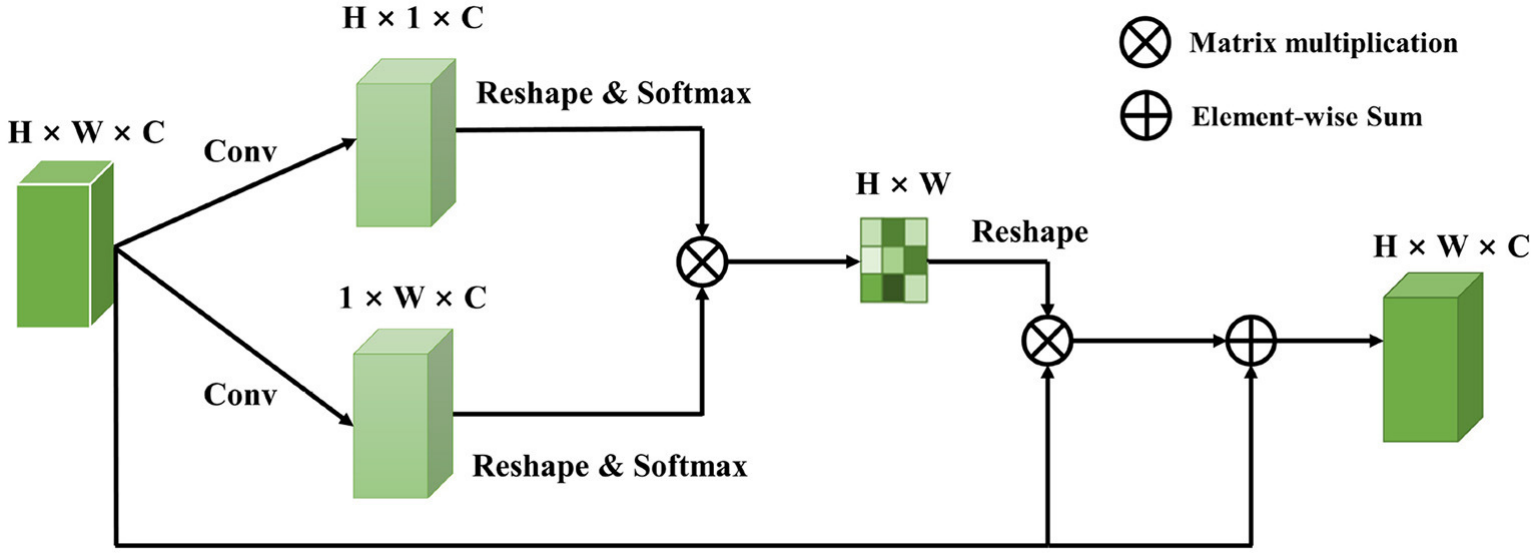
The detail of Dual Attentive Fusion Module.

Raw EEG signals contain spatial relationship among different channels and temporal dependency among different time points, which play an important role in feature classification. However, many studies (Kim and Choi, 2020; Tao et al., 2020; Zhang D. et al., 2020) suggest that features generated by traditional machine learning methods could not extract this information well. In 2017, the Transformer proposed by Vaswani et al. (2017) raised much attention in the natural language processing field. A transformer model is based on the self-attention module, which effectively focuses on the distinct features by assigning attention score to each feature and aggregating these scores. More and more work has introduced attention mechanism into the computer science field and achieved comparable performance. Recent work has focused on designing proper attention modules to adaptively explore attentive dynamics of EEG signals and focus on the most valuable information in brain-computer interface fields. Inspired by it, we propose a Dual Attentive Fusion Module which can take spatial and temporal attention into consideration in an interactive module. Our method can turn raw EEG signals into more discriminative features. More importantly, our method improves the accuracy of EEG signal classification. First, a filtering process is conducted on all EEG signals by implementing bandpass filter. Then, the proposed attention module is used to recode the EEG signals considering the spatial and temporal dimensions together. Finally, the features are fed into a convolutional neural network to make classification, and classification accuracy is considered as the final evaluation metric. The Dual Attentive Fusion Module is illustrated in Figure 2.

### 3.2. Dual attentive fusion module

As illustrated in Figure 1, an EEG signal **A**, is denoted as **A** ∈ ℝ^*H*×*W*^, where H is the number of electrodes and W is the number of time points. We first feed the data into a convolution layer to generate a new feature map **B**, where **B** belongs to ℝ^*H*×*W*×*C*^ and C = 1 denotes the number of feature map.

Then, to learn the spatial features of multi-channel EEG and explore the temporal features of different time points, we employ a self-attention mechanism in the EEG signals. In the spatial dimension, a self-attention operation turns channels into a probability distribution as weights and recodes the EEG signals based on the weights. In this way, an important feature would gain a higher weight than less important features. Therefore, we compress **B** to feature map **C** by a convolution layer, where **C** belong to ℝ^*H*×1×*C*^. A softmax activation function is applied to **C** to obtain the attention map of the spatial dimension:


(1) $$\begin{array}{l} a_{1}=\frac{exp\left(C_{i}^{T}v_{i}\right)}{\sum exp\left(C_{i}^{T}v_{i}\right)}, \end{array}$$

The attention vector *v*_*i*_ ∈ ℝ^*H*^ is randomly initialized and tuned by the above function during the training procedure. The softmax function makes sure the sum of weights is 1. The more similar feature representation of the two channels devotes to a more significant correlation between them.

Next, in the temporal dimension, to extract more discriminative temporal information, we also employ a self-attention operation to obtain a feature representation by perceiving global temporal features and assigning the weights according to the similarity of time points. Thus, a convolution layer is applied to compress **B** as **D**, which belongs to ℝ^1×*W*×*C*^. A softmax activation function is also applied to **D** to obtain the attention map of the temporal dimension:


(2) $$\begin{array}{l} a_{2}=\frac{exp\left(D_{i}^{T}w_{i}\right)}{\sum exp\left(D_{i}^{T}w_{i}\right)}, \end{array}$$

The attention vector *w*_*i*_ ∈ ℝ^*W*^ is randomly initialized and tuned by the above function during the training procedure. This attention map will focus on specific time points that are distinct from others. To enable matrix multiplication between *a*_1_ and *a*_2_, we reshape *a*_1_ and *a*_2_ as to ℝ^*H*×*C*^ and ℝ^*C*×*W*^, respectively.

Finally, a matrix multiplication is employed to obtain the spatial-temporal attention map a ∈ ℝ^*H*×*W*^ as:


(3) $$\begin{array}{l} a=a_{1}^{T}\cdot a_{2}, \end{array}$$

where *a*_1_ and *a*_2_ are spatial and temporal attention map of EEG signals, respectively. A dual attentive fusion feature representation is further generated by considering the spatial-temporal attention map as weights to recode EEG signals. Thus, a is reshaped to ℝ^*H*×*W*×*C*^, and we perform an element-wise matrix operation between a and **B**. The dual attentive fusion feature extracted by the Dual Attentive Fusion Module can be expressed as follows:


(4) $$\begin{array}{l} E=\sum \left(a_{i}\cdot B_{i}\right), \end{array}$$

where **E** ∈ ℝ^*H*×*W*×*C*^, *a*_*i*_ denotes the spatial-temporal attention map, and **B** represents the preprocessed EEG signals. In addition, a residual block (He et al., 2016) is applied between **E** and **B** to obtain the final dual attentive fusion feature as follows:


(5) $$\begin{array}{l} Z=W\cdot E+B, \end{array}$$

where W belongs to a learnable parameter, which is randomly initialized and is gradually updated during the training procedure. Equation (5) shows that the final feature of EEG signals is a weighted sum of the spatial-temporal features and original features.

### 3.3. Feature classification module

In this module, we employ a CNN, which is inherited the architecture of the EEGNet (Lawhern et al., 2018), to classify the features extracted from the previous module. A 2D convolution layer with a kernel size of (1, *K*_1_) is first applied to **Z** to capture temporal information in each electrode. Then, a depthwise convolution layer with a kernel size of (H, 1) is used for spatial feature extraction. An average pooling operation is followed to generate a coarser feature. Next, the separableConv2D with a kernel size of (1, *K*_2_) is used to obtain deeper temporal patterns across all electrodes. An average pooling operation is also followed to reduce dimension. It is worth noting that batch normalization (Ioffe and Szegedy, 2015) and exponential linear unit (Clevert et al., 2015) are followed by some convolution operations for feature standardization and nonlinear transformation. Finally, the deep feature extracted by CNN is flattened as a vector by a flatten layer. For binary classification, the output of dense layer is forwarded into a sigmoid function. For multi-class classification, the output of dense layer is forwarded into a softmax function. The final prediction is based on conditional probability, which is calculated by the loss function. The loss value guides the gradient descent and the backpropagation for the whole neural network. The structure of Feature Classification Module and its parameters are shown in Tables 1, 2.

**Table 1:** Architecture of feature classification module.

**Layer**	**Input**	**Filter**	**Kernel**	**Output**
Conv2D	(H, W, 1)	F1	(1, *K*_1_)	(H, W, *F*_1_)
BatchNorm	(H, W, *F*_1_)			(H, W, *F*_1_)
DepthwiseConv2D	(H, W, *F*_1_)	*F*_1_*D	(H, 1)	(1, W, *F*_1_*D)
BatchNorm	(1, W, *F*_1_*D)			(1, W, *F*_1_*D)
ELU activation	(1, W, *F*_1_*D)			(1, W, *F*_1_*D)
AveragePooling2D	(1, W, *F*_1_*D)		(1, *P*_1_)	(1, W/*P*_1_, *F*_1_*D)
SeparableConv2D	(1, W/*P*_1_, *F*_1_*D)	*F*_2_	(1, *K*_2_)	(1, W/*P*_1_, *F*_2_)
BatchNorm	(1, W/*P*_1_, *F*_2_)			(1, W/*P*_1_, *F*_2_)
ELU activation	(1, W/*P*_1_, *F*_2_)			(1, W/*P*_1_, *F*_2_)
AveragePooling2D	(1, W/*P*_1_, *F*_2_)		(1, *P*_2_)	(1, W/(*P*_1_*P_2_), *F*_2_)
Flatten	(1, W/(*P*_1_*P_2_), *F*_2_)			(W*F_2_)/(*P*_1_*P_2_)
Dense	(W*F_2_)/(*P*_1_*P_2_)			*N*

**Table 2:** Hyperparameter setting.

**Hyperparameter**	**II**	**III**	**IV-2a**	**IV-2b**
H	10	10	22	3
W	144	144	1,000	1,000
*F*_1_	8	8	8	8
*F*_2_	16	16	16	16
*K*_1_	72	72	64	64
*K*_2_	16	16	16	16
*P*_1_	4	4	4	4
*P*_2_	8	8	8	8
D	2	2	2	2
N	2	2	4	2

## 4. Experiments and results

In this section, we first describe the benchmark datasets used in this paper. Then, we demonstrate the model implementation details. Finally, we present the experimental results obtained by our method and other comparable approaches.

### 4.1. Dataset description

In our experiment, we use four public BCI competition datasets to evaluate the effectiveness of the proposed method. Among them, BCI Competition IV-2a (Tangermann et al., 2012) and BCI Competition IV-2b (Tangermann et al., 2012) are used for motor imagery classification. BCI Competition II Dataset IIb (Blankertz, 2010) and BCI Competition III Dataset II (Blankertz et al., 2008) are used for P300 detection. The detailed information of the four datasets is shown as follows.

#### 4.1.1. The BCI competition IV-2a dataset

The BCI competition IV-2a dataset, provided by Graz University, contains EEG signals from nine healthy subjects(A01-A09) and two sessions on different days for each subject. Each session consists of 288 trials of four different MI classes: imagining the movement of the left hand, the right hand, the feet, and the tongue. The signals are recorded by 22 electrodes at 250 Hz sampling frequency and bandpass filtered between 0.5 and 100 Hz. In this paper, as the same data division in the competition, we use the 288 trials of the first session as training and the 288 trials of the second session as testing. In each trial, we only use a 4 s temporal segment in our model, each sample can be represented as a 2D-matrix of 22 × 1, 000, in which 22 represents the number of electrodes and 1,000 represents the number of sample points.

#### 4.1.2. The BCI competition IV-2b dataset

The BCI competition IV-2b dataset is also collected from nine healthy people (B01–B09) at a sample rate of 250 Hz but only recorded from three electrodes placed at positions C3, Cz, and C4. For each subject, 720 trials from two MI tasks, including left-hand and right-hand movement imagination, are performed. There are five sessions for each individual. The first three sessions are for training, and the remaining two are for testing as the same data division in the competition. In this paper, The 4 s temporal segment of each trial is used as a sample, which can be represented as a 2D-matrix of 3 × 1, 000.

#### 4.1.3. BCI competition II—dataset IIb and BCI competition III—dataset II

**Figure 3:**
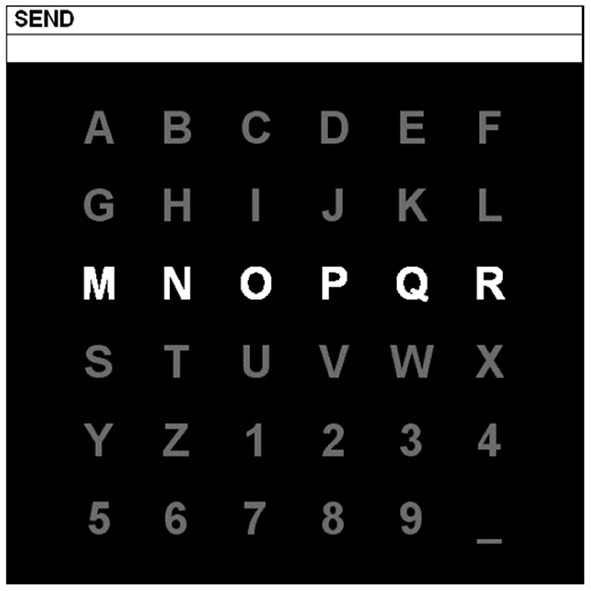
P300 speller paradigm.

Both datasets are offered by Wadsworth Center, New York State Department of Health. BCI Competition II—Dataset IIb is composed of a single subject data collected in three sessions containing 42 training and 31 testing characters. In BCI Competition III—Dataset II, there are two subjects: Subject A and Subject B. For each subject, the EEG signals are divided into a training set (85 characters) and a testing set (100 characters). In the experiments, the subject was presented with a 6 × 6 matrix of characters shown in Figure 3. In 1988, Farwell and Donchin developed this type of P300 speller paradigm (Farwell and Donchin, 1988). The user was asked to concentrate on the characters of a given word (one character at one time). All six rows and six columns randomly and successively intensified at 5.7 Hz. One row and one column out of these 12 intensive flashings contained the desired character. The sets of 12 intensifications were repeated 15 times for each character. The EEG data were bandpass filtered between 0.1 and 60 Hz and digitized at 240 Hz from 64 channels. In this paper, we choose 10 electrodes, including Fz, Cz, Pz, Oz, C3, C4, P3, P4, PO7, and PO8, in which the P300 signals are mainly generated. Due to a positive response around 300 ms after the onset of the stimulus in P300 ERP, we extract a time window of 600 ms after intensification onset as the input for each trial. With the collected frequency of 240 Hz, a trial can be denoted as a 10 × 144 data matrix.

### 4.2. Implementation details

In the motor imagery classification experiment, the model is implemented with the Keras framework and trained on Google online platform (Colab). The Adam optimizer (Kingma and Ba, 2014) with a learning rate of 0.001 is employed to minimize the cross-entropy loss function. The mini-batch size is set to 16, dropout regularization is 0.2, and the epoch is 1,000. Batch normalization is adopted to get better performance.

In the P300 detection experiment, the model is constructed with the Keras framework on Google online platform (Colab) and trained from scratch. The training procedure is performed by minimizing the binary cross-entropy loss function. It is guided by Stochastic Gradient Descent with Adam optimizer (Kingma and Ba, 2014). The learning rate is set as 0.001. The batch size is set to be 150, and the epoch is 300. Dropout regularization with 0.5 is applied in our model. Batch shuffling is implemented for better generalization.

### 4.3. Results on motor imagery datasets

#### 4.3.1. Comparison results

In order to evaluate the effectiveness of our proposed method, we compare it with other state-of-the-art methods, including FBCSP (Ang et al., 2012), CCSP (Kang et al., 2009), BOTDA (Peterson et al., 2021), EEGNet (Lawhern et al., 2018), ConNet (Zhang Y. et al., 2020), DEI (Zhang C. et al., 2021), DRDA (Zhao et al., 2020), DAJAN (Hong et al., 2021), and FTF (Zhang K. et al., 2021). Tables 3, 4 show the classification accuracies of each subject and the average accuracies of different methods on BCI IV-2a and IV-2b datasets, respectively.

**Table 3:** Classification accuracies (%) obtained with the dataset BCI competition IV-2a.

**Methods**	**Subject**									***Average* ± *SD***
	**A01**	**A02**	**A03**	**A04**	**A05**	**A06**	**A07**	**A08**	**A09**	
FBCSP	76.00	56.50	81.25	61.00	55.00	45.25	82.75	81.25	70.75	67.75 ± 13.73
CCSP	84.72	52.78	80.90	59.38	54.51	49.31	88.54	71.88	56.60	66.50 ± 15.13
BOTDA	80.43	55.83	80.90	57.64	55.39	62.79	70.23	81.92	80.68	69.38 ± 11.95
EEGNet	85.76	61.46	88.54	67.01	55.90	52.08	89.58	83.33	**86.81**	74.50 ± 15.23
ConNet	76.39	55.21	89.24	74.65	56.94	54.17	92.71	77.08	76.39	72.53 ± 14.24
DEI	81.85	53.71	81.25	66.67	57.97	63.72	84.48	79.70	79.92	72.14 ± 11.66
DRDA	83.19	55.14	87.43	75.28	62.29	57.15	86.18	83.61	82.00	74.70 ± 12.96
DAJAN	86.46	68.75	93.06	**85.42**	72.57	63.54	**95.49**	**85.76**	83.68	81.52 ± 10.94
FTF	83.27	57.24	91.94	66.67	76.45	**66.51**	86.28	83.39	82.58	77.15 ± 11.34
**DAFM**	**86.83**	**72.43**	**96.70**	74.56	**81.52**	64.65	91.69	85.60	84.84	**82.09 ± 10.02**

Highest values are highlighted in boldface.

**Table 4:** Classification accuracies (%) obtained with the dataset BCI competition IV-2b.

**Methods**	**Subject**									***Average* ± *SD***
	**B01**	**B02**	**B03**	**B04**	**B05**	**B06**	**B07**	**B08**	**B09**	
FBCSP	70.00	60.36	60.94	97.50	93.12	80.63	78.13	92.50	86.88	80.01 ± 13.85
CCSP	63.75	56.79	50.00	93.44	65.63	81.25	72.81	87.81	82.81	72.70 ± 14.72
BOTDA	61.40	55.92	54.78	88.93	92.67	73.71	71.98	86.35	79.18	73.88 ± 14.18
EEGNet	68.44	57.86	61.25	90.63	80.94	63.13	84.38	93.13	83.13	75.88 ± 13.33
ConNet	76.56	50.00	51.56	96.88	93.13	85.31	83.75	91.56	85.62	79.37 ± 17.25
DEI	70.18	62.04	71.74	90.23	86.08	75.70	89.66	87.39	85.71	79.86 ± 10.17
DRDA	81.37	62.86	63.63	95.94	93.56	**88.19**	85.00	95.25	**90.00**	83.98 ± 12.67
DAJAN	**83.44**	58.57	59.06	98.13	96.56	84.38	86.25	92.81	87.81	83.00 ± 14.64
FTF	78.07	68.16	73.04	96.74	95.24	84.86	92.67	92.17	85.71	85.18 ± 10.17
**DAFM**	70.18	**71.84**	**89.56**	**99.02**	**100.00**	73.71	**94.40**	**95.65**	88.98	**87.04 ± 11.95**

Highest values are highlighted in boldface.

We observe that the proposed method achieves the highest average classification accuracies of 82.09 and 87.04% on BCI IV-2a and IV-2b datasets, respectively. Regarding the experimental results of every subject, our method achieves accuracy above 70% except the A06 subject on both datasets. The best classification accuracy is obtained at the A03 and B05 subjects on BCI IV-2a and IV-2b datasets, respectively. Moreover, the standard deviation (SD) of our method is lower than that of other approaches on the BCI IV-2a dataset. On the BCI IV-2b dataset, the SD of our method is lower than that of other approaches except DEI and FTF. Generally, our method achieves the best results and has good stability on both MI datasets. The main reason that our method outperforms traditional methods is its nonlinear modeling ability which is the advantage of deep learning methods. Our method also has superior performance over other deep learning methods due to our proposed DAFM. Compared with the simple CNN models such as EEGNet and ConNet without dual attention mechanism, the proposed module improves the performance of the model by selectively amplifying valuable features and suppressing useless features based on the data-driven attentive scores.

**Figure 4:**
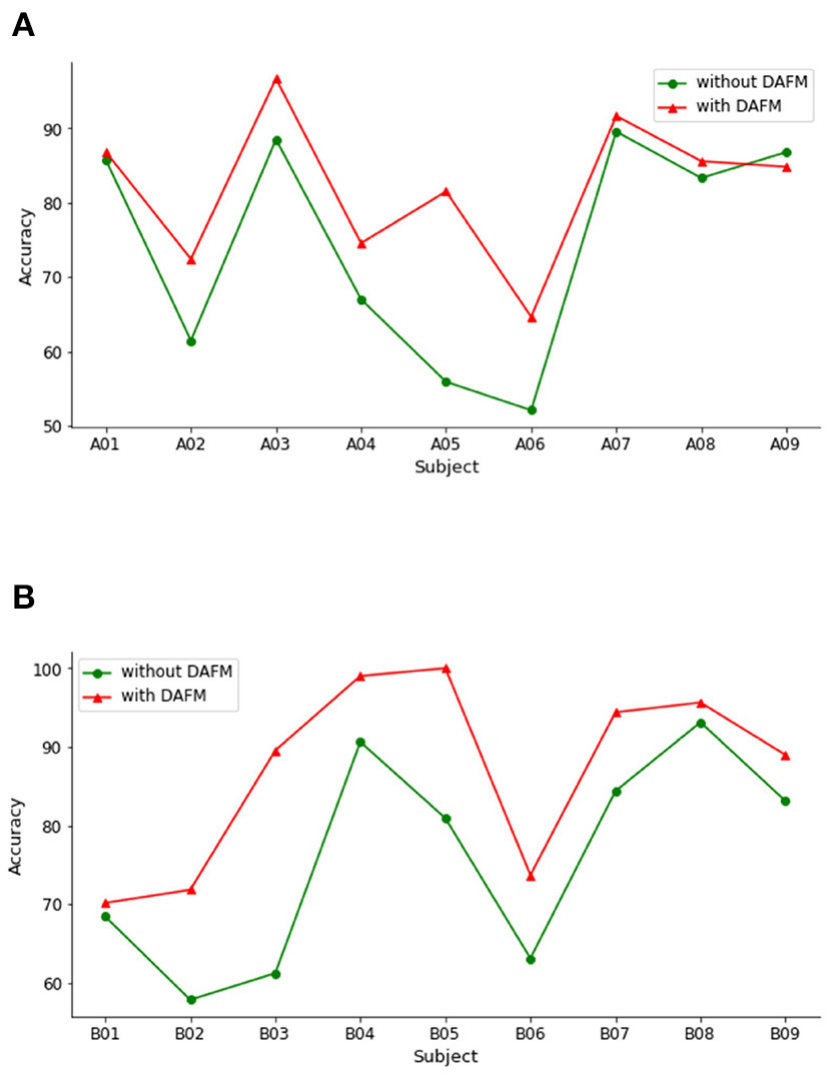
Classification accuracies across subjects with or without DAFM. **(A)** BCI IV-2a. **(B)** BCI IV-2b.

To evaluate the capacity of our method, we perform the classification experiments on BCI IV-2a and IV-2b datasets under both without DAFM and with DAFM, respectively. The classification accuracies on both datasets are shown in Figure 4.

On the BCI IV-2a dataset, DAFM has different influence on the classification accuracy for all subjects. The classification accuracies of eight subjects improve. Only the performance on subject A09 slightly decreases. As shown in Figure 4B, the performance of DAFM has a better performance across all subjects. These encouraging findings show that the DAFM is beneficial to MI classification and generate more discriminative feature regardless different individuals.

#### 4.3.2. Result of the confusion matrices

**Figure 5:**
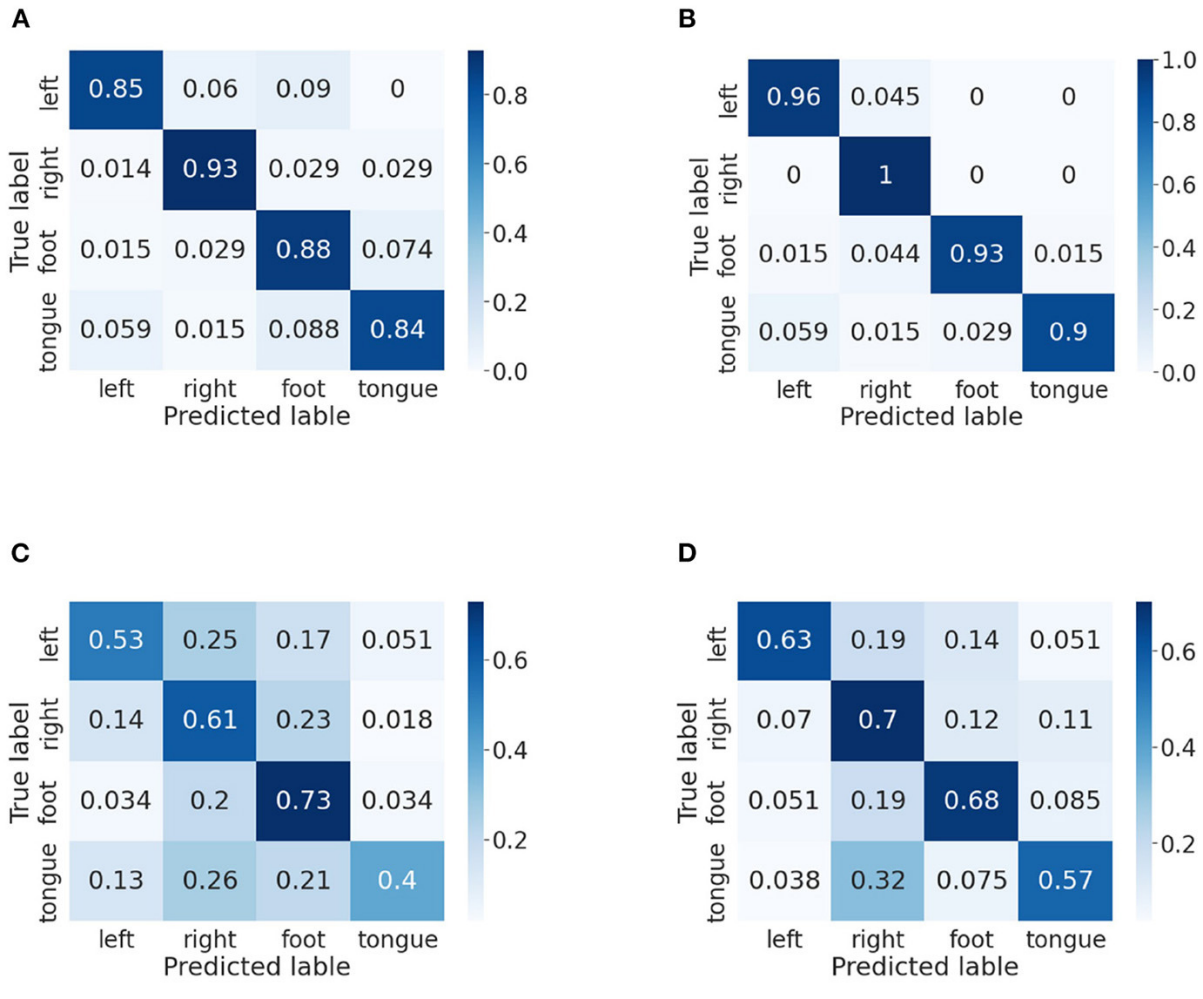
Confusion matrices of BCI competition IV-2a datasets. **(A)** A03 without our method. **(B)** A03 with our method. **(C)** A04 without our method. **(D)** A04 with our method.

**Figure 6:**
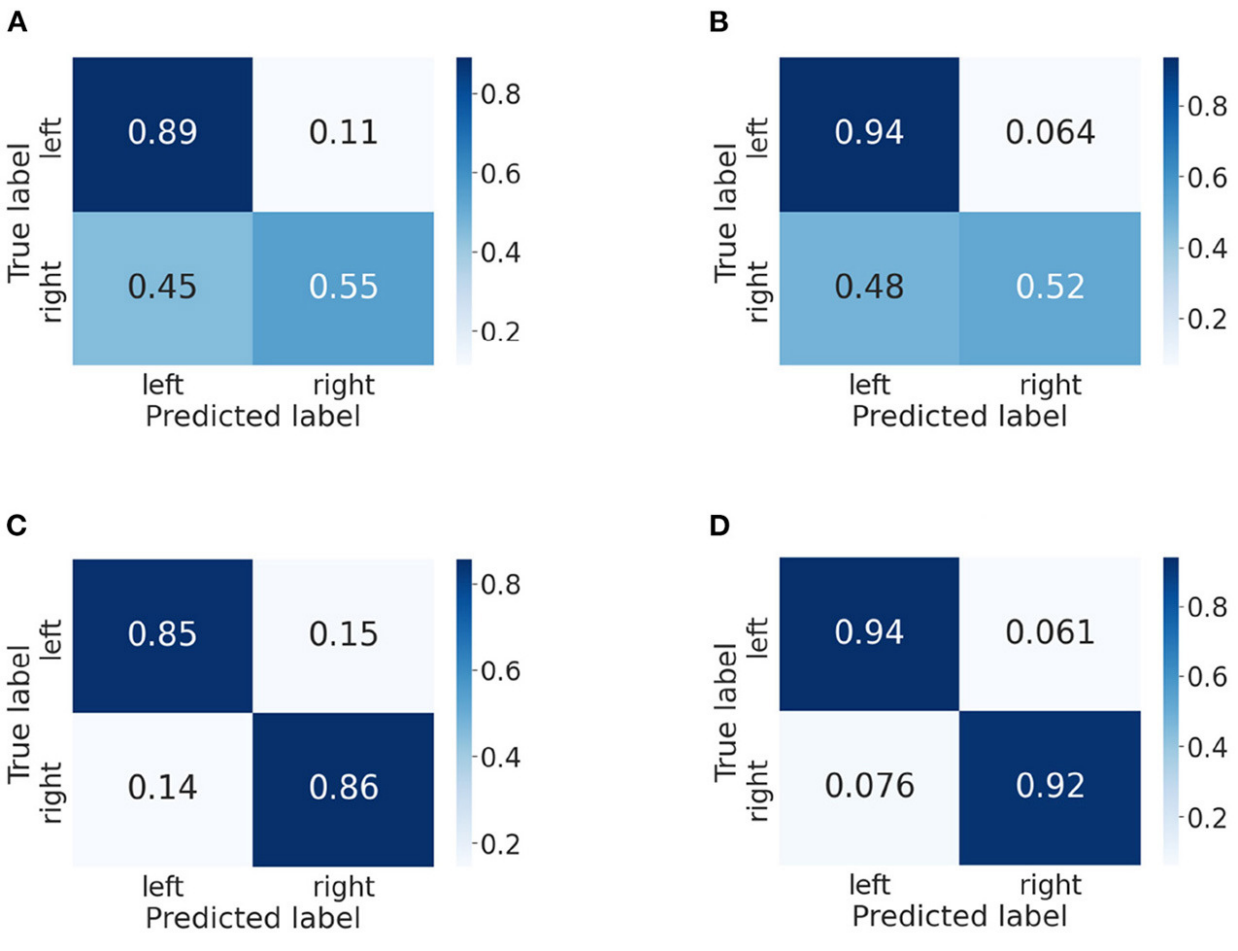
Confusion matrices of BCI competition IV-2b datasets. **(A)** B01 without our method. **(B)** B01 with our method. **(C)** B02 without our method. **(D)** B02 with our method.

In this part, we use confusion matrices to show the predictive outcome of our method in each class. Confusion matrices on BCI IV-2a and IV-2b datasets are presented in Figures 5, 6, respectively. The vertical axis represents the true label, and the horizontal one represents the predicted label. We randomly select two subjects on the BCI IV-2a dataset (i.e., A03 and A04) and BCI IV-2b dataset (i.e., B01 and B02).

First, on the BCI IV-2a dataset, for subject A03, we could observe that the left hand, right hand and foot are easier to be recognized than the tongue. By comparison between Figures 5A,B, in which Figure 5A does not use our method, we discover the classification accuracies of the four categories have improved significantly, which demonstrates that our proposed attention module is beneficial to the MI classification. Moreover, the gap between different classes has narrowed. For subject A04, it is obvious that the right hand and foot are easier to be recognized than the left hand and tongue. We could discover that the proposed method increases the classification rate between each class except the foot. However, without using our method, other categories are easily misclassified as foot, and by using our method, the misclassification rate has decreased a lot.

Then, we analyze the confusion matrices of the BCI IV-2b dataset, which has two classes. For subject B01, we can find that the left hand is much easier to be recognized than the right hand. Except this, we can see that the classification effect of the left hand significant improves though the right hand's classification rate decreases slightly. The comparison of Figures 6C,D indicates that our method improves the classification performance for subject B02 and reduces the misclassification rate of both classes.

### 4.4. Results on P300 datasets

#### 4.4.1. Comparison results

We perform a series of experiments on the BCI Competition II dataset and BCI Competition III dataset to further validate the effectiveness of our method. We compare the classification accuracies for our method with other state-of-the-art methods, including CNN1 (Cecotti and Graser, 2010), CNN3 (Cecotti and Graser, 2010), CNNR (Manor and Geva, 2015), BN3 (Liu et al., 2018), OCLNN (Shan et al., 2018), EEGNet (Lawhern et al., 2018), DeepConvNet (Zhang Y. et al., 2020), and ShallowConvNet (Zhang Y. et al., 2020). The experimental results on both datasets are shown in Tables 5, 6, respectively.

**Table 5:** Classification accuracy (%) on BCI competition II dataset.

**Method**	**Accuracy**
CNN1	89.70
CNN3	87.54
CNNR	89.52
BN3	88.26
OCLNN	87.37
EEGNet	91.49
DeepConvNet	91.49
ShallowConvNet	88.62
**Ours**	**93.64**

Highest values are highlighted in boldface.

**Table 6:** Classification accuracy (%) on BCI competition III dataset.

**Method**	**Subject**		**Average accuracy**
	**A**	**B**	
CNN1	85.25	89.08	87.17
CNN3	83.92	86.92	85.42
CNNR	84.83	89.17	87.00
BN3	84.67	90.33	87.50
OCLNN	85.33	90.58	87.96
EEGNet	86.92	91.75	89.34
DeepConvNet	87.00	90.50	88.75
ShallowConvNet	83.50	86.50	85.00
Ours	**87.50**	**92.50**	**90.00**

Highest values are highlighted in boldface.

We observe that the proposed method improves clearly compared to other approaches, with around 2.15% better than the second-best method on the BCI Competition II dataset. Table 6 shows that our method outperforms all the comparable methods, obtaining an average accuracy of 90.00% on the BCI Competition III dataset. Thus, the proposed method can achieve the best performance on both datasets. The experimental results demonstrate that DAFM provides a more accurate classification outcome for P300 detection task.

**Figure 7:**
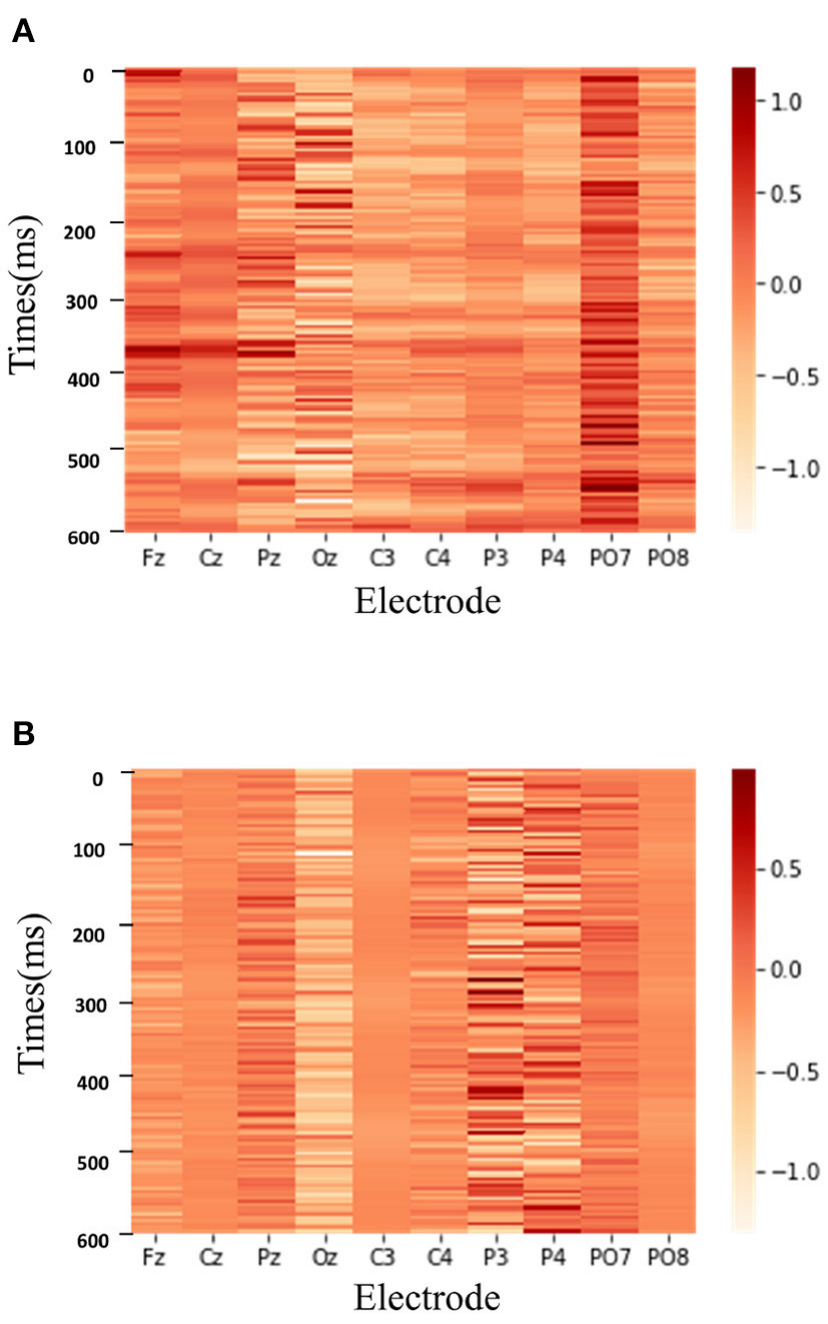
The two classes heatmap result of DAFM on the BCI Competition II dataset. **(A)** P300 signal. **(B)** Non-P300 signal.

To better demonstrate the role of the proposed method, we also exhibit the weighed features learned by our method. Figure 7 shows the two classes heatmap result of DAFM on the BCI Competition II dataset.

As shown in Figure 7, DAFM focuses on different ranges of EEG signals for P300 detection. The deeper the color is, the more attention the model pays to the corresponding part of EEG signals. When recognizing the P300 signal, the model has a high degree of attention around 300 ms time points due to a positive peak appearing after 300 ms of the stimulus in the P300 signal. In contrast, the feature map of the non-P300 signals has a more scattered appearance over time. It is illustrated that the proposed attention module can automatically learn the priority of different temporal points, which contributes to better performance.

#### 4.4.2. The training loss and testing accuracy of our method

**Figure 8:**
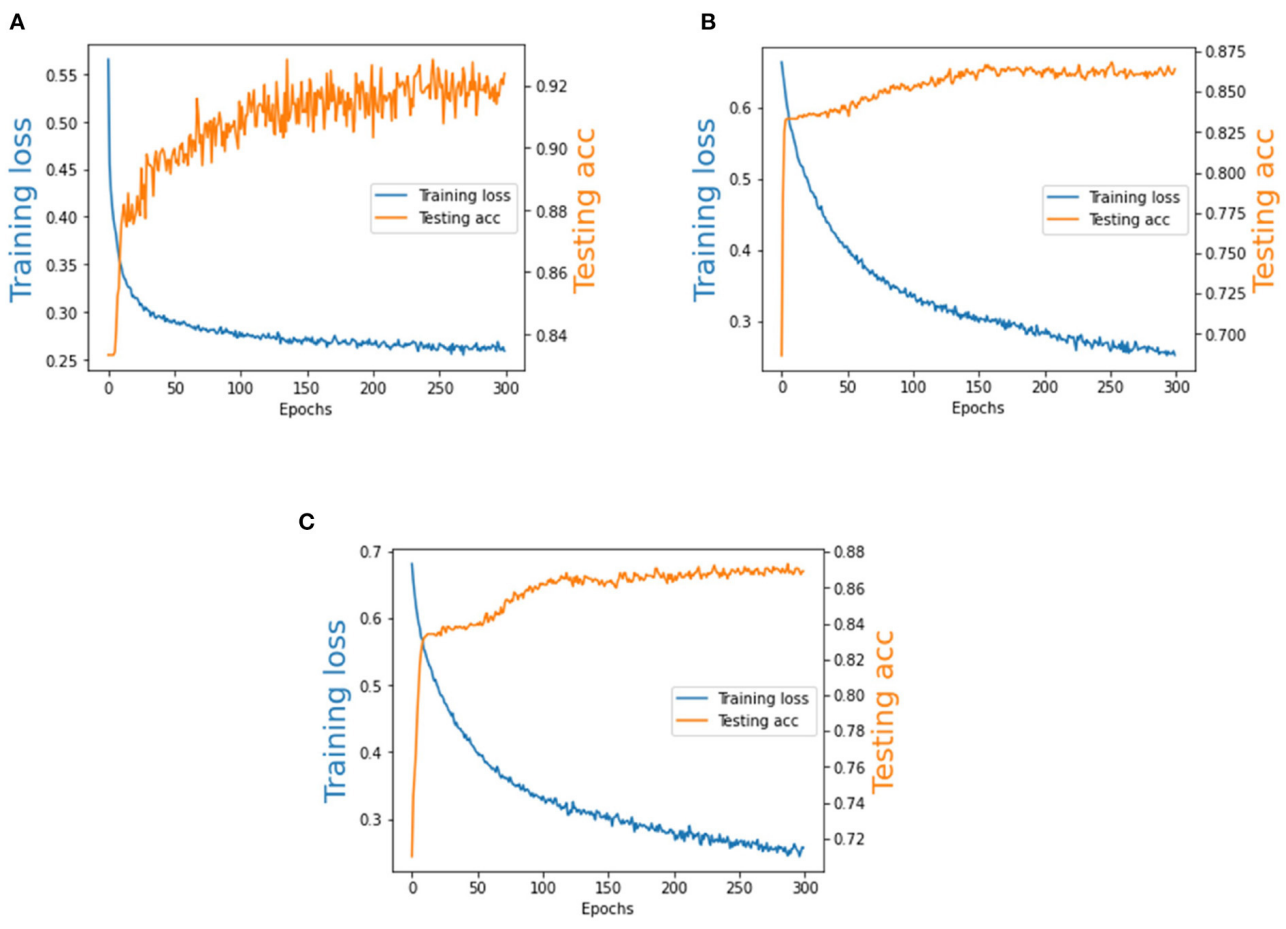
The training loss and testing accuracy of BCI Competition II dataset and BCI Competition III dataset. **(A)** BCI Competition II dataset. **(B)** Subject A of BCI Competition III dataset. **(C)** Subject B of BCI Competition III dataset.

We analyze the training loss and the testing accuracy of our method on the BCI Competition II dataset and BCI Competition III dataset. As is shown in Figure 8, the number of training epochs is 300. It can be observed that the testing accuracy increases quickly during the first 50 epochs and the training loss is generally stable after training about 100 epochs. Therefore, Our model exhibits a stable performance during the training procedure, and we observe that it converges quickly.

## 5. Conclusion

This study proposes a novel DAFM framework to effectively extract discriminative features from the EEG signals for different EEG-based classification tasks. It leverages an interactive attention module to generate the informative spatial-temporal features. The experimental results, conducted on four widely-used datasets, demonstrate that our method achieves superior performance to state-of-the-art and baseline methods. Our ablation experiments also confirm the effectiveness of our method. In summary, our method could be regarded as a potential approach to improve the performance of EEG-based BCI systems.

Due to a large amount of noise and artifacts in EEG signals, the proposed method can alleviate the interference of noise to a certain extent by focusing on useful information and ignoring useless information, but it cannot eliminate them. In the future, we plan to explore the more stable patterns of EEG signals using attention mechanism. Meanwhile, the proposed attention module will be extended to other tasks, such as image classification, semantic segmentation, etc.

## Data availability statement

The datasets presented in this study can be found in online repositories. The names of the repository/repositories and accession number(s) can be found at: http://www.bbci.de/competition/.

## Ethics statement

Ethical review and approval was not required for the study on human participants in accordance with the local legislation and institutional requirements. Written informed consent for participation was not required for this study in accordance with the national legislation and the institutional requirements.

## Author contributions

JH conceptualized the study, performed the majority of the experiments and analyses, made the figures, and wrote the first draft of the manuscript. XH, YD, KS, and NZ performed some experiments, updated the figures, performed the statistics, and edited the manuscript. All authors approved the submitted version.

## Funding

The work in this paper was supported in part by National Nature Science Foundation of China (No. 11901063).

## Conflict of interest

The authors declare that the research was conducted in the absence of any commercial or financial relationships that could be construed as a potential conflict of interest.

## Publisher's note

All claims expressed in this article are solely those of the authors and do not necessarily represent those of their affiliated organizations, or those of the publisher, the editors and the reviewers. Any product that may be evaluated in this article, or claim that may be made by its manufacturer, is not guaranteed or endorsed by the publisher.
